# Supplementary material for: Preoperative and perioperative factors that predict endothelial cell loss 1 year after uncomplicated Descemet membrane endothelial keratoplasty
Source: PLoS One. 2025 Dec 19;20(12):e0339346. doi: 10.1371/journal.pone.0339346 (PMC12716750; doi:10.1371/journal.pone.0339346)
Supplement: S1 File — (DOCX) [file pone.0339346.s001.docx]

# Supporting Information

# Supporting Figures

## S1 Fig. Endothelial cell loss at 12 months in eyes that underwent no, one, or multiple rebubbles (*n*=137). *P* values were determined by generalized linear regression analysis accounting for patient level.


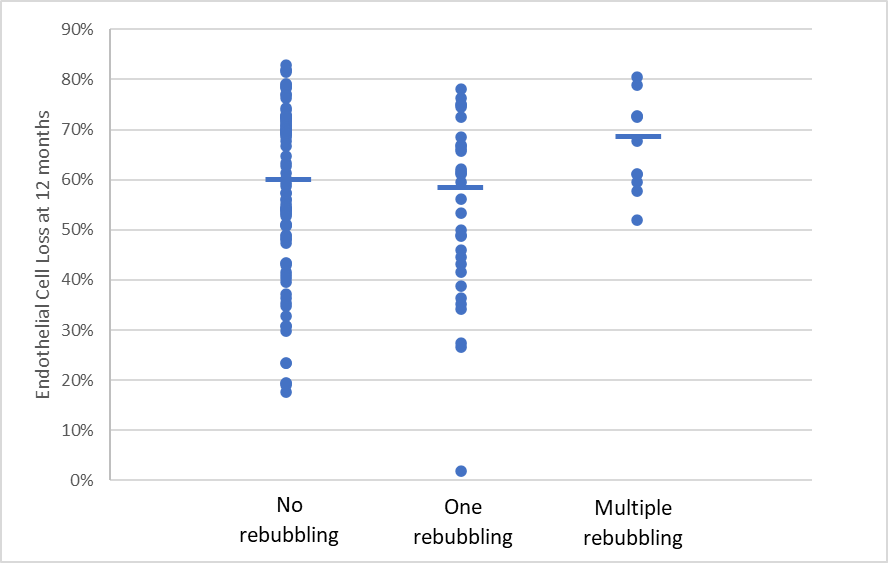


p=0.68

p=0.02

# Supporting Tables

## S1 Table. Preoperative, perioperative, and postoperative characteristics of the cohort (*n*=137)

| Characteristic | Missing values | Median (IQR) | *n* (%) |
| --- | --- | --- | --- |
| Age, years |  | 72 (67 – 78) |  |
| Female sex |  |  | 84 (61) |
| Operated eye on right |  |  | 75 (55) |
| Preop axial length, mm | 14 | 23.5 (22.8 – 24.6) |  |
| Preop anterior chamber depth, mm | 14 | 3.2 (2.8 – 3.7) |  |
| Preop lens thickness, mm | 70 | 4.8 (4.6 – 5.0) |  |
| Preop BSCVA, logMAR | 1 | 0.5 (0.4 – 0.7) |  |
| Preop CCT, µm |  | 614 (567 – 650) |  |
| Donor age, years |  | 75 (68 – 82) |  |
| Preop graft ECD, cells/mm² |  | 2560 (2420 – 2700) |  |
| General anesthesia |  |  | 137 (100) |
| Triple-DMEK |  |  | 69 (50) |
| Graft marking  With an F or F*  None |  |  | 106 (77)  31 (23) |
| SF6 use |  |  | 47 (34) |
| Operative time, min |  | 35 (30 – 40) |  |
| Rebubbling |  |  |  |
| None |  |  | 94 (69) |
| One |  |  | 32 (23) |
| Multiple |  |  | 11 (8) |
| BSCVA at 6 mo, logMAR |  | 0.17 |  |
| BSCVA at 12 mo, logMAR |  | 0.12 |  |
| BSCVA at 24 mo, logMAR |  | 0.08 |  |
| CCT at 6 months, µm | 68 | 540 (520 – 570) |  |
| CCT at 12 months, µm | 53 | 538 (516 – 569) |  |
| CCT at 24 months, µm | 64 | 537 (516 – 571) |  |
| ECD at 6 months, cells/mm² |  | 1200 (800 – 1500) |  |
| ECD at 12 months, cells/mm² |  | 1000 (730 – 1353) |  |
| ECD at 24 months, cells/mm² | 20 | 1000 (761 – 1300) |  |
| % ECL at 6 months |  | 53 (41 – 67) |  |
| % ECL at 12 months |  | 61 (48 – 72) |  |
| % ECL at 24 months | 22 | 62 (48 – 71) |  |

BSCVA, best spectacle-corrected visual acuity; CCT, central corneal thickness; DMEK, Descemet membrane endothelial keratoplasty; ECD, endothelial cell density; ECL, endothelial cell loss; IQR, interquartile range; mo, months; preop, preoperative; SF6, 20% sulfur hexafluoride.
