# Supplementary material for: Preoperative and perioperative factors that predict endothelial cell loss 1 year after uncomplicated Descemet membrane endothelial keratoplasty
Source: PLoS One. 2025 Dec 19;20(12):e0339346. doi: 10.1371/journal.pone.0339346 (PMC12716750; doi:10.1371/journal.pone.0339346)
Supplement: Attachment — Submitted filename: PLOS ONE Review.docx [file pone.0339346.s002.docx]

**Comments :**

The authors comprehensively evaluated potential risk factors for endothelial cell loss at 1 year after DMEK through extensive multivariate analyses. Although the manuscript is detailed and informative, I would like to highlight several major concerns below.

**Title**

In the short title, using **“Endothelial Cell Loss”** instead of **“ECL”** would be more meaningful and reader-friendly.

**Abstract**

Line 36 : In the phrase **“Fuchs- Endothelial…”**, it would be more appropriate to remove the hyphen between the words.

Line 47 : “Younger donor age tended to predict ECL (p=0.08).” The p-value is not below 0.05 and therefore not statistically significant; however, it has been interpreted as significant in the text. Normally, when the p-value exceeds 0.05, further post hoc analyses should not be performed. Nonetheless, a subsequent p-value of 0.02 is also reported. This section should be carefully reviewed for consistency and accuracy.

**Introduction :**

Line 63-64 : The use of the word **“loss”** twice in close succession disrupts the flow of the sentence.

Additionally, the **Introduction** section is rather lengthy; shortening it would improve readability and make the text more concise and engaging.

**Methods :**

Line 116 : should be ‘adhered’.

Line 202-205 : The postoperative topical steroid regimen has been mentioned; however, it requires further elaboration. It is unclear which specific agent is referred to as a “low-dose steroid.” The tapering strategy should be described more clearly, as ocular hypertension is a well-established postoperative risk following DMEK and is known to negatively affect endothelial cell density. Moreover, the rationale behind the continued use of topical steroids two to three times daily for five years should be clarified. Typically, the dosage is reduced to once daily by the end of the first year, and in some cases, clinicians even discontinue steroids after that period.

Line 210 : Was rebubbling always performed using air? Why was SF6 not utilized? It should be noted that SF6 may be preferred in certain situations where longer intraocular tamponade is desired—such as in eyes with previous glaucoma surgery, vitrectomized eyes, or those with scleral-fixated intraocular lenses.

Line 270-271 : The indications for rebubbling should be described in greater detail, particularly in the Materials and Methods section. According to the general literature, rebubbling is typically recommended when graft detachment exceeds one-third of the graft area. It is also unclear what is meant by “central detachment” in this context. For instance, if there is a 50% inferior detachment, is rebubbling not performed in such cases?

**Results :**

Line 262-277 : In the Results section, some data are presented in detail both in the text and in Tables 1 and 2. To avoid redundancy, if the data are already provided in the tables, the text should summarize them more concisely—focusing on statistical significance rather than repeating numerical values.

Line 285-288 : Here again, many data points were analyzed with further statistical tests despite p-values not being below 0.05. An explanation for this approach is necessary, as it deviates from standard statistical practice.

Line 315-316 : This part is not appropriate for the Results section; explanations supported by references should be presented in the Discussion section. Additionally, the abbreviation **GLM** has not been defined earlier in the text and should be clarified.

Line 317 and 327 :It should be ‘SF6 use WAS associated with..’

Line 332 : It should be **“was”** instead of **“is”**, as the text generally uses the past tense. Overall, there is inconsistent use of present and past tense throughout the manuscript, and it would benefit from a thorough English language review.

Line 325-349 : The section beginning with **“Multivariate analyses exploring relationships between variables in terms of ECL”** reads more like a Discussion than a Results section and requires substantial revision. For the entire manuscript, the Results section should focus solely on reporting findings, avoiding interpretations or references to previous studies. Explanations and contextual discussion of the results should be reserved for the Discussion section.

Line 364-366 : “Older donor age promoted ECL when operative time was long and also increased ECL somewhat when SF6 was used.” According to the Abstract, **young donor age** was listed as a risk factor. This should be checked for consistency with the Results and Discussion sections to ensure that the finding is accurately reported and properly contextualized.

Line 367-375 : Again, although this is part of the Results section, it contains interpretative comments that belong in the Discussion section.

**Discussion**

Line 479-480 : “To conclude this section, rebubbling is inconvenient for both the patient and the surgeon, and it might increase ECL.” Although it may cause discomfort, a detached graft will result in prolonged corneal edema and delayed visual recovery. Therefore, it can be misleading to conclude that rebubbling should be avoided

Line 534-535 : The p-value here appears somewhat high to be considered **“almost significant.”**

Line 606 : The use of multivariate analyses undoubtedly adds value to the study. However, it appears that a large number of parameters were assessed. Were **all potential variables that could be significant for ECL** included in the multivariate analysis, or was the evaluation limited to a smaller set of data chosen based on consistency with the literature?

In addition to the above comments, one of the most important limitations of the study is the lack of evaluation of **patients’ preoperative ocular characteristics**. Although the patients are described as uncomplicated, no details are provided. For example, lens status, prior vitrectomy, presence of glaucoma, or history of previous glaucoma surgery should be reported. These characteristics should be incorporated into the study results and assessed using appropriate correlation analyses.

The manuscript would also benefit from a **comprehensive English language review**, particularly regarding the use of articles (e.g., “the”).

Furthermore, the manuscript is very long—specifically, the Discussion section exceeds ten pages. For improved readability, it is recommended to **shorten the text**, avoid repetition, and ensure that points appropriate for the Discussion are not presented in the Results section.
